# The Latest Insights into Adipokines in Diabetes

**DOI:** 10.3390/jcm8111874

**Published:** 2019-11-05

**Authors:** Won Kon Kim, Kwang-Hee Bae, Sang Chul Lee, Kyoung-Jin Oh

**Affiliations:** 1 Metabolic Regulation Research Center, Korea Research Institute of Bioscience and Biotechnology (KRIBB), 125 Gwahak-ro, Yuseong-gu, Daejeon 34141, Korea; wkkim@kribb.re.kr (W.K.K.); khbae@kribb.re.kr (K.-H.B.); 2 Department of Functional Genomics, KRIBB School of Bioscience, Korea University of Science and Technology (UST), 217 Gajeong-ro, Yuseong-gu, Daejeon 34141, Korea

**Keywords:** diabetes, adipokines, biomarkers, diagnosis, prediction, therapeutic targets

## Abstract

The Special Issue “Pathogenetic and Therapeutic Significance of Adipokines in Diabetes” focused on adipokines as shared diagnostic biomarkers and therapeutic targets for both obesity and type 2 diabetes. Experts discussed the pathological role of adipokines in their studies associated with diabetes. It provided new insights into the role of adipokines in diabetes. In this commentary and review, these studies will be summarized and the novel roles of adipokines will be discussed. This will also confirm the role of adipokines as biomarkers for diagnosis and prediction, and as therapeutic targets of diabetes and its related pathogenic phenomena.

Obesity and type 2 diabetes are considered as global epidemic and pandemic diseases [1,2,3]. The majority of patients with type 2 diabetes are obese, suggesting a close association between type 2 diabetes and obesity. The term “diabesity” was coined by Dr. Francine Kaufman to explain the strong relationship between obesity and type 2 diabetes [4]. These conditions are caused by energy imbalances in peripheral metabolic organs, such as the liver, muscles, and adipose tissues. To maintain energy homeostasis, these organs communicate and exert complementary effects on each other. Secretory peptides or proteins from metabolic organs facilitate the communication with each organ. These secretory factors are classified and described as myokines from the muscle, adipokines from adipose tissues, and hepatokines from the liver, which are all necessary for metabolic adaptation in obesity and type 2 diabetes [5].

Among these secretory factors, adipokines are more notable because of their important role in providing the shared pathogenesis of obesity and type 2 diabetes [6,7]. Obesity is defined as a condition of excessive accumulation of body fat. Its development and severity are directly influenced by adipokines [8]. Excess adiposity, which is associated with the dysregulated expression of adipokines, triggers adipocyte dysfunction, inflammation, and peripheral and whole-body insulin resistance [9]. Adipokines are categorized into “proinflammatory adipokines” and “anti-inflammatory adipokines”; the former promotes inflammation and insulin resistance, whereas the latter plays a protective and beneficial role [5,10]. The imbalance between proinflammatory and anti-inflammatory adipokines leads to pathogenic changes. As a typical example, patients with obesity and type 2 diabetes exhibit altered adipokine profiles, leading to profound metabolic risk and alterations in insulin sensitivity [5,7].

The Special Issue “Pathogenetic and Therapeutic Significance of Adipokines in Diabetes” aimed at providing new pathophysiological insights into adipokines in diabetes (Figure 1). This editorial will review six papers published as one review and five research articles in the Special Issue [7,11,12,13,14,15]. As a review article, Lee et al. recently defined the range of adipokines as adipose-derived secretion factors [7]. Adipose tissues are categorized into two types: white adipose tissue (WAT) and brown adipose tissue (BAT), according to their morphology and function [16,17]. Secretory bioactive molecules from WAT and BAT are referred to as “adipokines” and “batokines”, respectively [7,18]. In their study, the authors mainly sought to update and report new adipokines and batokines [7]. Additionally, they reported that exosomal miRNAs are released from adipose tissues and regulate various metabolic events in an endocrine manner [7]. Therefore, they described that adipose-derived signals, as a broad concept of adipokines, involve adipokines, batokines, and exosomal miRNAs [7]. Further, they suggested that adipose-derived factors would be biomarkers for the diagnosis and prediction of the risk for both obesity and type 2 diabetes [7]. As a totally new perspective, this study provided an opportunity to establish a novel role of adipokines as an endocrine organ.

Other authors also discussed the altered expression pattern of adipokines in their experimental sets or clinical samples associated with diabetes [11,12,13,14,15]. Yagmur et al. investigated the diagnostic and clinical relevance of plasma C1q/TNF-related protein 1 (CTRP1) in critically ill patients [11]. CTRP1, belonging to the CTRP superfamily, is mainly secreted by adipose tissues and is involved in many pathological conditions, such as inflammation and insulin resistance [19,20]. Critically ill patients are at a higher risk of acquiring a spectrum of metabolic disorders, including diabetes, and various inflammatory conditions, such as obesity, sepsis, and immune defense. They also found that there is an increase in the levels of plasma CTRP1 in critically ill patients with sepsis and diabetes, and they demonstrated that elevated plasma CTRP1 levels can be considered as a biomarker for the prediction of sepsis and diabetes in critically ill patients [11]. Therefore, they assumed that CRTP1 could be a promising biomarker of inflammatory response and metabolic disturbance, including sepsis and type 2 diabetes, in critically ill patients.

Shih et al. assessed the relationship between adiponectin and aortic arterial stiffness (AS) in patients with type 2 diabetes [12]. Adiponectin is a well-known adipokine that has anti-diabetic, anti-inflammatory, and anti-atherogenic properties [5,7,21]. Decrease in the levels of circulating adiponectin helps predict the risk of type 2 diabetes, abdominal obesity, and insulin resistance [22,23,24], and is associated with atherosclerotic cardiovascular diseases [25,26,27,28]. Therefore, they hypothesized that circulating adiponectin levels might be related to aortic AS in type 2 diabetes [12]. As a result, the authors demonstrated that low circulating adiponectin levels can be considered as an independent indicator to predict the risk of aortic AS in patients with type 2 diabetes.

Lin et al. evaluated the association between secretogranin III (SCG3) and metabolic syndrome (MetS) [13]. SCG3 is a member of the granin family found in various endocrine and neuroendocrine cells [29,30,31]. It affects glucose homeostasis by regulating insulin secretion from pancreatic β-cells [32,33]. MetS is characterized by compensatory hyperinsulinemia and insulin resistance [34,35]. Therefore, SCG3 and MetS seem to be linked with each other. MetS is a cluster of at least three of the five following medical conditions: abdominal obesity, high blood pressure, high fasting glucose, high serum triglycerides, and low serum high-density lipoprotein. The authors found that circulating SCG3 levels were higher in individuals with MetS and those with high fasting glucose, abdominal obesity, or high serum triglycerides, one of the conditions referring to MetS [13]. Therefore, serum SCG3 levels would act as a biomarker for the prediction of the risk of MetS and would independently function as a potential target in regulating glycemia.

Ko et al. investigated the effects of aerobic exercise training on hepatic expression and functions of asprosin in streptozotocin (STZ)-induced type 1 diabetic rats [14]. Unlike type 2 diabetes that is characterized by insulin resistance, type 1 diabetes results from the destruction of insulin-producing pancreatic β-cells. Asprosin is an adipokine that is released from WATs [36,37]. Increased circulating asprosin levels are associated with increased blood glucose levels, whereas reduced circulating asprosin levels lower blood glucose levels and improve diabetic phenotypes [36,37,38]. Furthermore, asprosin has been reported to be closely related to hepatic glucose production [36]. However, whether aerobic exercise training affects hepatic asprosin expression and function in type 1 diabetes remains unclear. Ko et al. demonstrated that STZ increased hepatic asprosin and activated cAMP/PKA pathway that promotes hepatic glucose production. The effects of STZ were ameliorated by exercise in STZ-induced type 1 diabetic rats [14]. Additionally, STZ reduced phosphorylated AMPK and AKT levels, which were alleviated by exercise. Furthermore, STZ increased TGF-beta expression, whereas exercise suppressed its expression. These results suggested that aerobic exercise affects hepatic asprosin-dependent PKA/AMPK/TGF-β pathway in type 1 diabetes. Collectively, aerobic exercise training decreases hepatic asprosin levels, resulting in the amelioration of diabetic parameters in type 1 diabetic rats.

Kimber-Trojnar et al. demonstrated the potential role of fatty acid binding protein 4 (FABP4) as a biomarker for gestational diabetes, diagnosed in women with glucose intolerance beyond 24–28 weeks of gestation [15]. Circulating adipokines have pathophysiological significance in metabolic diseases, such as obesity, type 2 diabetes, cardiovascular disease, and hypertension [5,7,39,40]. However, the pathological role of adipokines in gestational diabetes is lacking. Women with a previous history of gestational diabetes and excessive gestational weight gain are at a higher risk of acquiring type 2 diabetes, obesity, and various metabolic diseases in the future [41,42]. FABP4 is a member of the intracellular lipid-binding protein family that transports fatty acids. FABP4 expression is gradually increased during adipocyte differentiation; therefore, it is considered as an adipocyte differentiation marker [43]. Recently, it has been reported that FABP4 is secreted from adipose tissues and functions as an adipokine. For example, adipose FABP4 promotes insulin secretion from β-cell and has an insulinotrophic function [44]. Kimber-Trojnar et al. described that there was an increase in the serum FABP4 levels in women with gestational diabetes in the early postpartum period and were positively related to serum leptin levels [15]. Therefore, these findings support that elevated serum FABP4 levels might be used as a predictive marker for future diseases in women with a history of gestational diabetes. However, the pathophysiological relevance requires further studies.

Additionally, recent reports have described that the disturbance in adipokines, as an endocrine organ, contribute to the development and aggravation of nonalcoholic fatty liver disease (NAFLD), i.e., the main cause of chronic liver diseases [45,46,47]. NAFLD mainly occurs due to abnormal uptake of free fatty acids derived from the lipolysis of adipose tissues [48]. Like other pathogenic conditions, NAFLD is also associated with type 2 diabetes and obesity [48,49,50]. In case of adiponectin and leptin, the classical adipokines and their roles in NAFLD pathogenesis have been well documented [45,46,47]. Furthermore, efforts to reveal the relationship between NAFLD and proinflammatory and anti-inflammatory adipokines have already been made. However, the types and roles of adipokines are constantly being updated, as shown in a review article of this Special Issue [7]. Therefore, there is a need to establish novel roles of adipokines in NAFLD to make them an interesting issue associated with adipokines.

Collectively, diabetes is accompanied by several pathogenic phenomena, such as obesity, sepsis, aortic AS, and cardiovascular diseases (Figure 1). Therefore, the role of pathologically disturbed adipokine profiles in various pathogenic conditions should be systemically understood to provide clues for the diagnosis, prediction, and treatment of diabetes.

## Figures and Tables

**Figure 1:**
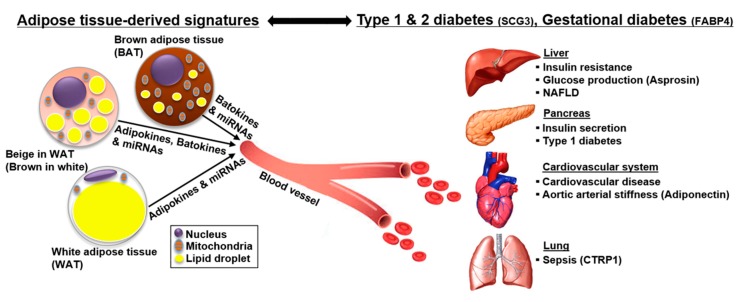
The role of adipokines in diabetes and its related pathogenic phenomena. Adipokines play a critical role in maintaining systemic energy homeostasis. Diabetes is accompanied by several pathogenic phenomena, such as obesity, sepsis, aortic arterial stiffness (AS), and nonalcoholic fatty liver disease (NAFLD). Pathologically disturbed adipokines in diabetes can act as biomarkers for the diagnosis and prediction of the risk for diabetes and its related pathogenic conditions. Further, understanding the role of adipokines in various pathogenic conditions will provide clues for the treatment of diabetes. Abbreviations: SCG3, secretogranin III; FABP4, fatty acid binding protein 4; CTRP1, C1q/TNF-related protein 1.
